# Effects of thermal fluctuations on biological processes: a meta-analysis of experiments manipulating thermal variability

**DOI:** 10.1098/rspb.2022.2225

**Published:** 2023-02-08

**Authors:** Margaret A. Slein, Joey R. Bernhardt, Mary I. O'Connor, Samuel B. Fey

**Affiliations:** ^1^ Department of Biology, Reed College, Portland, OR 97202, USA; ^2^ Department of Zoology and Biodiversity Research Centre, University of British Columbia, Vancouver, British Columbia, Canada V6T 1Z4; ^3^ Department of Ecology and Evolutionary Biology, Yale University, 165 Prospect Street, New Haven, CT 06520, USA; ^4^ Yale Institute for Biospheric Studies, PO Box 208118, New Haven, CT 06520, USA; ^5^ Department of Integrative Biology, University of Guelph, Guelph, Ontario, Canada N1G 2W1

**Keywords:** thermal variability, thermal performance, environmental variation, phenotypic plasticity

## Abstract

Thermal variability is a key driver of ecological processes, affecting organisms and populations across multiple temporal scales. Despite the ubiquity of variation, biologists lack a quantitative synthesis of the observed ecological consequences of thermal variability across a wide range of taxa, phenotypic traits and experimental designs. Here, we conduct a meta-analysis to investigate how properties of organisms, their experienced thermal regime and whether thermal variability is experienced in either the past (prior to an assay) or present (during the assay) affect performance relative to the performance of organisms experiencing constant thermal environments. Our results—which draw upon 1712 effect sizes from 75 studies—indicate that the effects of thermal variability are not unidirectional and become more negative as mean temperature and fluctuation range increase. Exposure to variation in the past decreases performance to a greater extent than variation experienced in the present and increases the costs to performance more than diminishing benefits across a broad set of empirical studies. Further, we identify life-history attributes that predictably modify the ecological response to variation. Our findings demonstrate that effects of thermal variability on performance are context-dependent, yet negative outcomes may be heightened in warmer, more variable climates.

## 1. Introduction

Thermal variability is ubiquitous and can be a driving force affecting performance at the organism or population level [1], species coexistence at the community level [2], and the extent of species' geographical ranges and their responses to climate change [3,4]. Thermal variability has recently received considerable attention among ecologists because of its potential to influence multiscale biological processes [1,2,5], and the likelihood that global climate change has and will continue to impact the nature of environmental variation [6–8]. Both empirical and theoretical research demonstrates that explicitly considering the consequences of thermal variability can yield quantitatively and qualitatively different predictions about how populations and communities respond to thermal gradients, compared to predictions based on solely mean temperature changes [3,4,9]. Thus, meeting the grand challenge of accurately projecting future ecological responses to climate change across a range of ecological systems and biological contexts to inform policy and conservation decisions [10] requires a detailed understanding of the specific consequences of thermal variation.

Despite the importance of understanding the biological consequences of thermal variability, we lack evidence of general patterns to guide expectations across the many taxa, domains of variation, and biological responses that exist. At its most fundamental level, variability can either increase or decrease measures of organismal or population performance, simply because the thermal dependence of the underlying biological response is nonlinear [3,5,11]. For example, depending on how the thermal regime an organism experiences relates to its critical thermal limits (i.e. temperatures which, when exceeded for an extended duration, are lethal or sublethal), variation could result in either detrimental or beneficial outcomes [12,13]. Accordingly, the biological consequences of thermal variation have been interpreted as highly context-dependent [14–17]. Given the ongoing challenge of relating projections of organismal performance to climate change, filling the knowledge gap of whether performance in varying environments increases or decreases performance compared to constant laboratory conditions would represent an important practical advance in climate change ecology research [4,7].

To bridge this knowledge gap, we consider whether variation experienced in the past (i.e. prior to when an assay is performed) or the present (i.e. during the period where an assay is performed) is expected to have positive or negative consequences for the traits and rates of organisms and populations. We anticipated the ecological consequences of responses to variation in the past versus the present to differ, as predicted by the beneficial acclimation hypothesis (BAH) [18]. Under the BAH, organismal performance during a thermally fluctuating period that follows a period of constant temperature may be reduced due to the time lag required to become acclimated to new thermal conditions. However, organisms that can acclimate rapidly, relative to the pace of environmental change, may be able to mitigate such detrimental periods [19]. Conversely, under a ‘jack of all trades, master of some’ framing (*sensu* [20]), thermal variation experienced in the past may have positive consequences on performance during subsequent periods of sustained temperature exposures (e.g. cold hardening [21]), because prior acclimation to thermal fluctuations may be generally beneficial for performance in a novel, sublethal thermal environment. By contrast, organisms only experiencing variability in the present, with no previous exposure in the past, may experience decreases in performance once fluctuations commence.

The traits organisms possess may also predictably modify the outcome of experienced variation. Large organisms may be able to more effectively buffer variability in the present [22–24], as increased thermal inertia can reduce the extent of fluctuations in body temperature amidst environmental fluctuations. Conversely, smaller organisms can have a reduced ability to regulate body temperature to the same fluctuations and thus may be more susceptible to decreases in performance amidst rapid thermal shifts. Additionally, the age of an organism may modify its thermal tolerance and response to experienced environmental variation as life stage can be associated with both increases or decreases in the tolerance to thermal extremes [25,26]. Thus, the anticipated ecological responses to thermal variability based on past or present exposure *a priori* indicate sufficient nuance is required to accurately predict when variation may enhance or diminish performance across broad scales.

**Figure 1.**
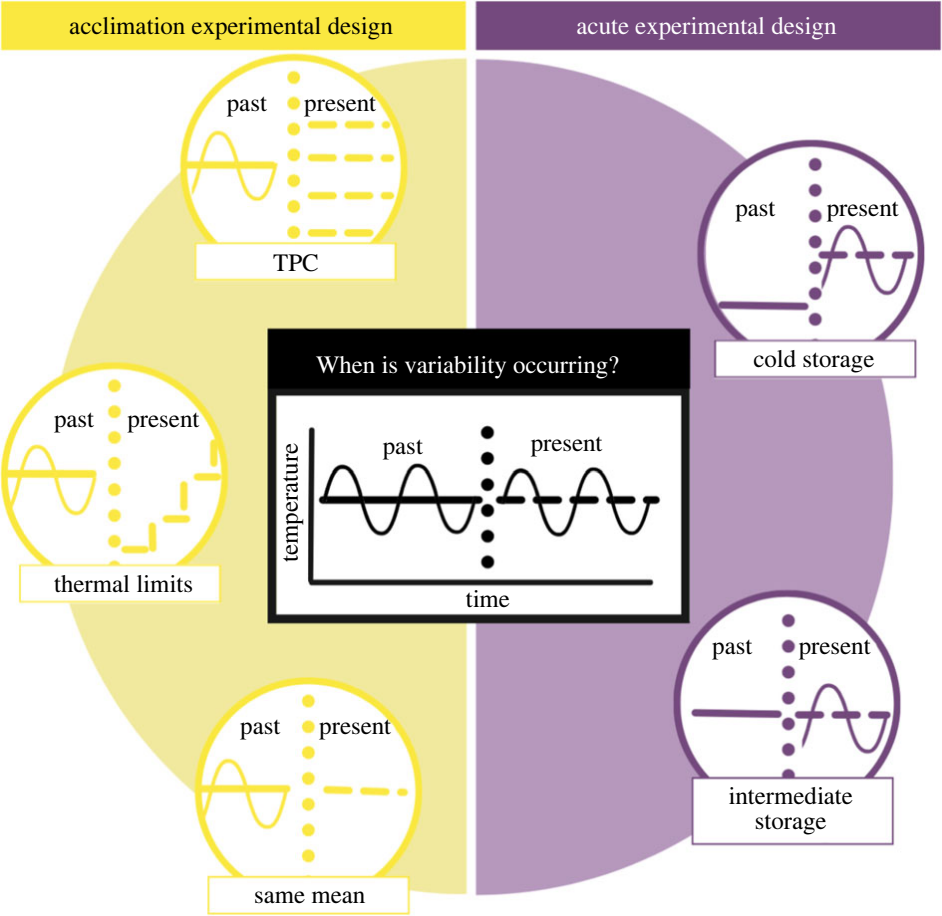
How thermal variability is integrated into two experimental designs based on when variability occurs. Acclimation experimental designs (yellow) feature studies focusing on the effects of variability in the past (solid line) with examples of how present environmental conditions differ across studies. Acute experimental designs (purple) feature studies focusing on the effects of variability in the present (broken line) with examples of how present environmental conditions differ across studies. Horizontal broken line indicates distinction between past and present. (Online version in colour; print version shows acclimation (light grey) and acute (dark grey) experimental designs.)

While recent synthetic efforts highlight the importance of variability across ecological time scales and levels of biological organization [27], it remains unclear how thermal variability impacts ecological processes across taxa, life stages, and a variety of experimental designs. Here, we leverage the existence of two common, contrasting experimental designs: experiments manipulating thermal variability experienced in the past (e.g. acclimation experimental designs) and thermal variability experienced in the present (e.g. acute experimental designs), when biological responses in these variable environmental conditions are measured relative to constant conditions (figure 1). We conducted a meta-analysis to test the following hypotheses regarding thermal variability using a range of taxa, treatments and ecosystems: (1) organisms benefit from experiencing variable environmental conditions in the past (acclimation), while organisms are disadvantaged from experiencing variation in environmental conditions solely in the present without prior acclimation (acute); (2) the response of organisms to variability under either exposure regime (acclimation or acute) is such that responses at both higher mean temperatures and higher amplitude variation exacerbate the negative effects of variability; and (3) organismal responses to variability depend on the traits of size and age, both of which are associated with how well the organisms can buffer environmental conditions, and whether responses measured indicate beneficial or detrimental biological effects.

## 2. Methods

### (a) Systematic literature review

To understand how thermal variability affects performance, defined as physiological or demographic rates or states, we conducted two systematic literature searches of the effects of thermal variation during acclimation and acute conditions. Our first search, conducted on 14 November 2020 using the ISI Web of Science (WOS) database with the search terms: AK = ((temperature OR thermal) NEAR (vari* OR fluc*)) AND SU = (Life Sciences & Biomedicine) yielded 176 results. To increase sample size and decrease publication bias, we conducted a second systematic literature search on 3 June 2021 using the SCOPUS database with the search terms: KEY (thermal performance curve OR thermal fluct* OR thermal vari* OR temperature vari* OR fluctuating temperatures OR thermal regime AND (ecology OR physiology)), which yielded 405 results. There were 43 papers returned in both WOS and SCOPUS searches.

### (b) Inclusion criteria

**Figure 2.**
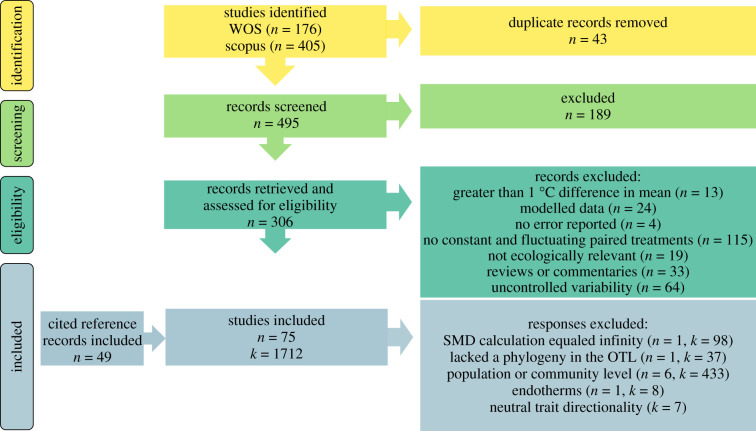
Preferred Reporting Items for Systematic Reviews and Meta-Analyses (PRISMA) diagram showing how records were assessed, screened, and included in the meta-analysis. ‘n’ refers to studies and ‘k’ refers to effect sizes.

We screened abstracts and titles from both searches for inclusion using the 0.4.1 version of the *revtools* R package [28] and excluded 189 studies (figure 2). We then assessed eligible studies (*n* = 306) and excluded studies that lacked a constant and fluctuating treatment (*n* = 115), did not feature a consistent, controlled fluctuation pattern (e.g. pulse press, multiple stochastic cold exposures, etc.) (*n* = 64), were reviews, commentaries, or perspectives (*n* = 33), were theoretical or modelling studies (*n* = 24), were not biologically relevant (e.g. engineering, chemical studies, etc.) (*n* = 19), lacked reported error measurements (*n* = 4), lacked extractable or comparable data (*n* = 4), and featured more than 1°C difference between the mean temperatures in constant and fluctuating treatments (*n* = 13). For studies to meet these inclusion criteria, the experimental design had to be explicitly focused on thermal variability. Subsequently, we conducted a cited reference search from the remaining eligible studies and included an additional 49 studies. In total, we included 75 studies with 1712 effect sizes (figure 2) (see electronic supplementary material, table S2 for a list and description of studies included). All studies included involved ectothermic organisms. We excluded any population or community-level responses as well as species with unresolved phylogenies or that were not identified to the species level in the Open Tree of Life database.

### (c) Data extraction

From the studies that met our inclusion criteria, we extracted mean response values, any measure of variance (standard deviation (s.d.), standard error of the mean (s.e.m.), etc.), and sample size from tables and figures using Webplotdigitizer [29]. Any studies that reported error as s.e.m. were converted to s.d. by multiplying s.e.m. by the square root of the sample size. Further, if studies reported findings using medians and the interquartile range (IQR), and we could confirm the data to be approximately normally distributed, we estimated the mean based on the reported median, and the s.d. to be the IQR divided by 1.5 [30]. If any extracted values were missing sample sizes or variances, the points were automatically excluded via the meta-analysis software *metafor* (v. 3.0.2, [31]). Additionally, we collected aspects of experimental design (experiment type, duration, etc.), thermal regime (mean temperature, fluctuation range, etc.) as well as life-history traits (age, size) and response metrics (trait directionality, see Analysis and Hypothesis Testing for definition) to investigate potential mechanisms mediating responses to thermal variability.

### (d) Analysis and hypothesis testing

Effect sizes were calculated using the escalc function in the 3.0.2 version of the package *metafor* [32] in R 4.1.0 [33]. *Metafor* builds on the functionality of older meta-analysis packages (*meta*, etc.) by allowing for greater flexibility in model types (mixed and random effects models) [31]. We specified the effect size metric of interest to be the standardized mean difference (SMD) because this is a common calculation used to compare two groups and it standardizes the responses across studies to reduce heterogeneity and bias [31]. We defined the SMD as the difference in mean reported empirical observations (response) between thermally variable and constant experimental treatments (SMD = (Mean_variable_ – Mean_constant_/Pooled Standard Deviation) × Correction factor J) [31] such that a negative SMD indicates that performance in variable conditions was worse than performance in constant environments and vice versa for a positive SMD. By using SMD, we were able to include a diverse array of responses such as development period, egg size, morphology and movement velocity (electronic supplementary material, table S1). This diversity of responses makes interpreting the biological consequences of a directional effect difficult. For example, a positive or larger effect size may mean a beneficial effect of the experimental treatment (i.e. fecundity) or a detrimental effect (i.e. increased cortisol levels) for organisms. The use of effect sizes is not intended to fully capture the biological consequences of a response variable but instead to compare responses across different experiments. As such, we attempted to capture some of the likely biological impacts by converting effect sizes associated with detrimental effects to a negative value by multiplying these SMDs by −1 [34].

We used a multilevel mixed-effects meta-analytic model via the rma.mv function in the 3.0.2 version of the package *metafor* [32] in R 4.1.0 [33] to model variation in effect sizes:

2.1 $$\;\begin{array}{rl} y_{i\;} & =\;\beta_{0}+\;\beta_{1}(\mathrm{Experiment}\;{\mathrm{type}}_{ijk})+\;\beta_{2}(Tm_{ijk})+\beta_{3}(Tf_{ijk})+\beta_{4}(Tm_{ijk}*Tf_{ijk})+\beta_{5}({\mathrm{age}}_{ijk})\\ & \;+\beta_{6}({\mathrm{size}}_{ijk})+\beta_{7}({\mathrm{ecosystem}}_{j})+\beta_{8}(\mathrm{trait}\;{\mathrm{directionality}}_{k})+\beta_{9}(Ts_{ijk})+\beta_{10}({\mathrm{duration}}_{ijk})\\ & \;+\beta_{11}(\mathrm{publication}\;\mathrm{bia}s_{ijk})+\beta_{12}(Tm_{ijk}\times \mathrm{Experiment}\;\mathrm{typ}e_{ijk\;})+\beta_{13}(Tf_{ijk}\times \mathrm{Experiment}\;\mathrm{typ}e_{ijk})\\ & \;+\;\beta_{14}({\mathrm{age}}_{ijk}\times \mathrm{Experiment}\;{\mathrm{type}}_{ijk})+\beta_{15}({\mathrm{size}}_{ijk}\times \mathrm{Experiment}\;\;\mathrm{typ}e_{ijk})\\ & \;+\beta_{16}(\mathrm{trait}\;{\mathrm{directionality}}_{ijk}\times \mathrm{Experiment}\;{\mathrm{type}}_{ijk\;})+\mu_{k}+\;\mu_{i}\;+\;\mu_{j}+\;\varepsilon_{ijk}. \end{array}$$

This equation for the full model includes sets of terms that allow us to test our hypotheses. All models included random effects for the *k*th response type (development time, egg size, growth rate, etc.), the *j*th study, and the *i*th phylogenetic group (μ*_k_*, μ*_j_*, μ*_i_* respectively) (electronic supplementary material, table S1). To test hypothesis 1, we considered versions of the model (equation (2.1)) with and without terms for Experiment type*_ijk_*. Experiment type*_ijk_* allows us to contrast effects of past versus present exposure to variability by indicating the timing in which each unique SMD was reported relative to the experimental organisms' exposure to thermal variability, with levels of *acute* in which responses were measured during exposure to thermal variability (figure 1, right), and *acclimation* for responses measured after thermal variability had been experienced in the past (figure 1, left). Within a single study (single reference) *j*, SMDs for multiple response types *k* (e.g. growth rate and individual size) and phylogenetic groups *i* (e.g. *Limnodynastes tasmaniensis*, *Limnodynastes peronii*) could be recorded. To test hypothesis 2, we compared models with and without terms for mean temperature (*Tm_ijk_*,°C), magnitude of fluctuation in the thermally varying experimental treatment (*Tf_ijk_*, max T – min T in°C), and their interaction (Tm_ijk_*Tf_ijk_). To test hypothesis 3, we compared models with and without terms for ecological traits: age class (Age*_ijk_* with levels larval, juvenile, adult), size class (Size*_ijk_* with levels small, medium, large), and whether increasing effect size would have a positive or negative effect on growth, survival, or fitness (trait directionality*_k_*). We noted other aspects of the studies that potentially could modify differences in biological responses between control and variable treatments as estimated by SMD. We compared models with and without these potentially confounding factors: temperature (°C) to which organisms were acutely exposed (for acclimation studies) or at which organisms were stored before assays (for acute studies) (secondary temperature, *Ts_ijk_* ), the type of ecosystem an organism was from (Ecosystem*_j_* with levels terrestrial or aquatic/marine), and the amount of time (days) spent in experimental conditions (Duration*_jk_*). We included a phylogenetic correlation as a random effect to account for differences between phylogenetic groups. To estimate phylogenetic correlation, we used the Open Tree of Life v3.0.12 R package *rotl* (v3.0.12) to construct a phylogenetic tree and subsequent correlation matrix of species relatedness based on the species that were in our dataset [35,36]. Random effects were normally distributed with a mean of 0. We normalized continuous variables (mean temperature, fluctuation range, secondary temperature) around their respective grand means. We log transformed duration to achieve a normal distribution. We also calculated and included a covariance matrix for studies that shared control treatments to further minimize bias in our model [37].

We used maximum likelihood to estimate the performance of each model in our sets [31]. We compared models using Akaike information criterion small-sample equivalent (AICc), and we selected the model with the lowest AICc value and delta AICc as our final model (see electronic supplementary material, table S4 for details on candidate models). In the event of multiple models with delta AICc values less than 2, we would model average, using the model.avg function in the MuMIn package [38].

Once we identified the best model, we tested our hypotheses in the following way. First, we considered there to be support for any terms retained in the best model. To evaluate weight of evidence in support of terms related to our hypotheses, we examined the sign, magnitude, and confidence estimate for the relevant *β* coefficients (equation (2.1)). To evaluate some hypothesized effects, coefficients needed to be added across multiple model terms [39]. For example, to estimate the effects of organism size when variability was experienced in the past (acclimation) or present (acute), we summed the coefficient estimates for the intercept (*β*0) and the estimate for size class 2 (*β*6) for acute experiments because experiment type was a binary variable with acute specified as the reference level; we summed the coefficient estimates for the intercept (*β*0), the estimate for experiment type acclimation (*β*1), the estimate for size class 2 (*β*6) and the estimate for the interaction between experiment type and size (*β*15) for acclimation experiments. We inferred strength of evidence (strong, moderate and weak) based on the magnitude of coefficients (or, summed coefficients) retained in the best model. We inferred strong evidence for an effect based on coefficient estimates of *β <* 0.8 following Sullivan & Feinn [40]. We inferred moderate evidence for an effect based on coefficient estimates of *β <* 0.5. We inferred weak evidence for an effect based on coefficient estimates of *β* < 0.2 [40]. We inferred statistical significance based on whether the confidence intervals spanned zero, given that the term was retained in the best model and the known limitations of *p*-values in mixed effects models [41]. We inferred no support for terms not retained in the best model.

We observed no strong (*r* > 0.7) correlation between likely colinear factors (e.g. age and size) (see SI methods). We further assessed heterogeneity at all levels of our models to confirm sources of variance across each dataset and computed *I*^2^ values at each level based on [42] (electronic supplementary material, table S4). We did not include an *R*^2^ statistic because it was not appropriate for this multilevel, mixed-effects model [43].

### (e) Publication bias

To understand whether our searches and curated databases contributed significant publication bias to our results, we visualized effect sizes using funnel plots of the residuals [44]. We acknowledge that funnel plots may experience distortion due to multiple effect sizes from the same study with the same sample size [44]. To further assess publication bias we followed the procedures outlined by Nakagawa *et al.* [45] to assess the significance of funnel asymmetry which could suggest publication bias. In addition, we calculated Rosenthal's fail-safe number, which estimates the number of missing studies averaging a *z*-value of zero needed to make effect sizes statistically insignificant [46,47].

## 3. Results

We screened 495 candidate papers and cited references to identify 75 studies that met our inclusion criteria with 1712 effect sizes. In all, this dataset included observations on 82 species, all ectotherms, and generally small, young organisms, with the common traits measured being: CTmin, walking speed, and longevity (electronic supplementary material, table S1, table S6). The most represented taxa were: *Tetranychus*, *Drosophila*, *Plutella,* and *Pelodiscus.* Additionally, because the vast majority of responses focused on organismal responses (81% overall), we excluded any population or community-level responses (21 studies, 433 effect sizes) from analyses (see electronic supplementary material, table S1 for categories of moderators) because they were underrepresented and unevenly distributed between acute versus acclimation experiment types. We excluded any traits that did not have a clear positive or negative biological implication for large effect sizes (i.e. thermal preference, etc.) (7 effect sizes). We also excluded 3 species that had either unresolved phylogenies or were not identified to the species level in the Open Tree of Life database (3 studies, 37 effect sizes). Ecosystem was not retained our best model, thus there is not a reported estimate for the effect of variability across ecosystem types.

Our final, best model (which involved model averaging two models (see electronic supplementary material, table S3 for model descriptions)) included secondary temperature, duration, publication bias, with an interaction between experiment type and the following moderators: age, size, trait directionality, mean temperature, and fluctuation range. This best model also featured an interaction between mean temperature and fluctuation range. Funnel plots did reveal marginal asymmetry in positive and negative residuals (see electronic supplementary material, figure S1) in addition to sampling variance appearing with a significant effect from our model coefficient estimates, all of which suggest potential publication bias. However, these results are not a true estimate of publication bias and while they suggest potential bias, our estimation of a large Rosenthal's Fail-Safe number of 260819 suggests the converse.

**Figure 3.**
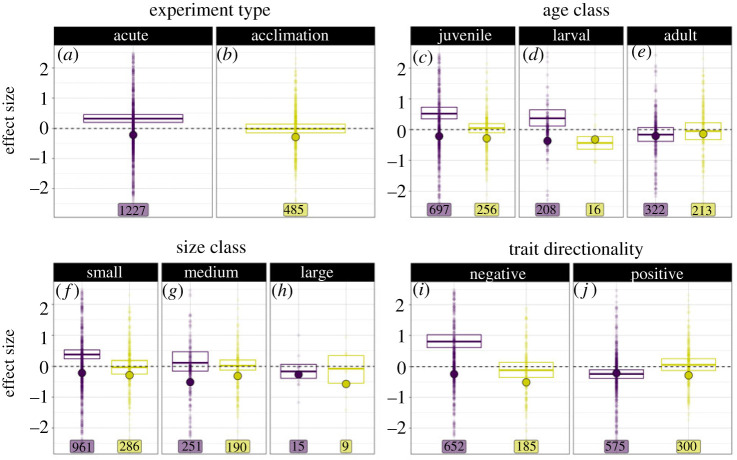
Raw effect sizes (SMD) between −2.5 to 2.5 for categorical variables relating to experiment type (*a*,*b*), size class (*c*,*d*,*e*), age class (*f*,*g*,*h*), and trait directionality (*i,j*). Colour corresponds to experiment type, labels correspond to sample size of effect sizes across categorical moderators and experimental design, horizontal solid lines represent the mean effect sizes for each level with 95% confidence intervals, large coloured dots represent coefficient estimates from the best model output (table 1). See electronic supplementary material, figure S2 for the full distribution of effect sizes (−15.3 to 31.6) and electronic supplementary material, figure S3 for the full distribution of effect sizes panelled by categorical variable and experiment type. (Online version in colour; print version shows acclimation (light grey) and acute (dark grey) experimental designs.)

We found that thermal variability experienced in the present (acute experimental designs) was associated with moderate, negative effect sizes, based on the retention of experiment type in the best model (electronic supplementary material, table S3) and the overall intercept value of *β*_0_=−0.213 (table 1; figure 3*b*; electronic supplementary material, table S5), though these effects were not statistically significant given that confidence intervals spanned zero. Additionally, we found minimal between-study heterogeneity (*I*^2^_study_ = 0.02) in our analysis. Variability experienced in the past (the acclimation experimental design) had even stronger negative, statistically significant effects on biological responses (*β*_0_ + *β*_1_ = −0.284) (table 1; figure 3*a*; electronic supplementary material, table S5). These negative coefficients suggest that thermal variability experienced in that past was associated with larger and more negative biological responses. Responses we identified as having negative biological consequences were associated with a moderate negative, statistically significant effect on SMD when variability was experienced in the present (*β*_0_ + *β*_8_ = −0.242) (figure 3*i*; table 1) and an even more negative, statistically significant effect when experienced in the past (*β*_0_ + *β*_1_ + *β*_8_ + *β*_16_ = −0.267) (figure 3*i*; table 1). These results indicate that thermal variation tends to heighten potentially unfavourable outcomes rather than decrease advantageous outcomes.

**Table 1.** Model output from the average of two best models. Statistical significance, indicated by confidence intervals that do not overlap 0, is indicted by an asterisk symbol next to the estimate.

coefficient	estimate	std. error	*Z* value	CI lower	CI upper
intercept	−0.213	1.351	0.158	−2.860	2.433
fluctuation magnitude	−0.009*	0.002	5.893	−0.013	−0.006
mean temperature	−0.061*	0.002	37.836	−0.065	−0.058
experiment type – acclimation	−0.071*	0.033	2.195	−0.135	−0.008
secondary temperature	0.000	0.001	0.170	−0.002	0.002
duration (log10 transformed)	−0.047*	0.032	1.488	−0.110	0.015
age—larval	−0.153	0.027	5.732	−0.205	−0.100
age—adult	0.007	0.032	0.231	−0.055	0.070
size—medium	−0.296	0.262	1.131	−0.810	0.217
size—large	−0.051	0.960	0.053	−1.934	1.831
trait directionality—negative	−0.029*	0.015	2.009	−0.058	−0.001
publication bias	1.491*	0.187	7.985	1.125	1.857
fluctuation magnitude × mean temperature	−0.002*	0.000	5.677	−0.003	−0.001
experiment type—acclimation × fluctuation magnitude	0.031*	0.004	7.922	0.023	0.038
experiment type—acclimation × mean temperature	0.057*	0.003	19.745	0.052	0.063
experiment type—acclimation × age—larval	0.117	0.138	0.848	−0.084	0.443
experiment type—acclimation × age—adult	0.137*	0.123	1.117	0.038	0.383
experiment type—acclimation × size—medium	0.269*	0.073	3.687	0.126	0.412
experiment type—acclimation × size—large	−0.234	1.123	0.208	−2.435	1.968
experiment type—acclimation × trait directionality—negative	−0.196*	0.053	3.732	−0.299	−0.093

**Figure 4.**
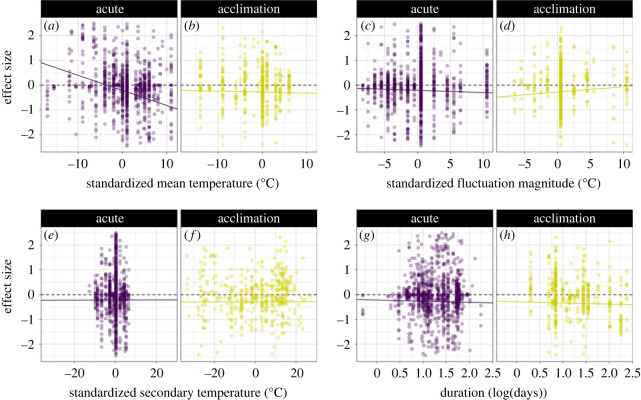
Scaled raw effect sizes (SMD) across standardized mean temperature (°C) (*a*,*b*), fluctuation range (°C) (*c*,*d*), secondary temperature (°C) (*e*,*f*) and duration of experimental conditions (log10(days)) (*g*,*h*), coloured and panelled by experiment type. Raw scaled effect sizes for mean temperature, fluctuation range and secondary temperature are all centred around their respective means, as we transformed these moderators accordingly in our model. Each point represents a scaled effect size (filtered to a range from −2.5 to 2.5). Samples sizes of scaled effect sizes input into the full model: acclimation (*n* = 485), acute (*n* = 1227). Solid lines represent the slope and intercept from the best model outputs and dashed lines indicate an effect size of 0. For duration and secondary temperature, the slopes for both experiment types were the same as we didn't include an interaction between duration and experiment type. See electronic supplementary material, figure S2 for the full distribution of effect sizes (−15.3 to 31.6) and electronic supplementary material, figure S4 for the full distribution of effect sizes panelled by categorical variable and experiment type. (Online version in colour; print version shows acclimation (light grey) and acute (dark grey) experimental designs.)

Thermal variability experienced in the present was associated with larger negative effect sizes at higher mean temperatures (figure 4*b*). With each 1°C increase in mean temperature, relative to 24°C, SMD decreased by 0.061 (*β*_2_ = −0.061) (table 1; electronic supplementary material, table S5), indicating a strong, statistically significant detrimental effect of increasing mean temperature on SMD at a mean temperature of 34°C (SMD = −0.6). Variability experienced in the past was associated with negative effect sizes at higher mean temperatures (figure 4*a*), though with each 1°C increase in mean temperatures, relative to 24°C, SMD only decreased by 0.004 (*β*_2_ + *β*_12_ = −0.004) (table 1; electronic supplementary material, table S5). This subtle negative effect of variability precipitates weak, statistically significant detrimental effects of increasing mean temperature on SMD at a mean temperature of 34°C (SMD = −0.04).

Similarly, variability experienced in the present was associated with negative effects sizes at higher fluctuation magnitudes (figure 4*d*), such that with each 1°C increase in fluctuation magnitude, relative to 10°C, effect sizes declined by 0.01 (*β*_3_ = −0.009) (table 1; electronic supplementary material, table S5), indicating a weak, statistically significant detrimental effect of increasing fluctuation magnitude on SMD at a fluctuation amplitude of 20°C (SMD = −0.1). Alternatively, variability experienced in the past was associated with positive effect sizes at higher fluctuation magnitudes (figure 4*c*), such that with each 1°C increase in fluctuation magnitude, relative to 10°C, effect sizes declined by 0.022 (*β*_3_ + *β*_13_ = 0.022) (table 1; electronic supplementary material, table S5), indicating a moderate, statistically significant positive effect of increasing fluctuation magnitude on SMD at a fluctuation magnitude of 20°C (SMD = 0.22). Lastly, the statistically significant interaction between mean temperature and fluctuation range suggests that at higher temperatures and fluctuation ranges, the negative effects of variability may be even further amplified as fluctuation magnitude and mean temperature simultaneously increase (*β*_2_ + *β*_3_ + *β*_4_ = −0.072) (table 1; electronic supplementary material, table S5, figure S5).

For variability experienced in the present, we observed a moderate, statistically significant negative effect of age on SMD suggesting that larvae responded more strongly to thermal variability than juveniles or adults, inconsistent with hypothesis 3 (figure 3*c–e*). Responses to variability experienced in the present decreased for the larval (*β*_0_ + *β*_5 larval_ = −0.366) (figure 3*d*; table 1; electronic supplementary material, table S5) and adult age classes (*β*_0_ + *β*_5 adult_ = −0.206) (figure 3*e*; table 1; electronic supplementary material, table S5) relative to juveniles (*β*_0_ = −0.213) (figure 3*c*; table 1, electronic supplementary material, table S5). For variability experienced in the past, we observed a weak, statistically significant negative effect of the adult age class on SMD (*β*_0_ + *β*_1_ + *β*_5 adult_ + *β*_14 adult_ = −0.154) (figure 3*e*; table 1; electronic supplementary material, table S5) in contrast to a moderate, but not statistically significant, negative effect of larval age class on SMD (*β*_0_ + *β*_1_ + *β*_5 larval_ + *β*_14 larval_ = −0.32) (figure 3*c*; table 1; electronic supplementary material, table S5).

Additionally, we detected a moderate, statistically significant negative effect of variability experienced in the past associated with medium sized organisms on SMD (*β*_0_ + *β*_1_ + *β*_6 medium_ + *β*_15 medium_ = −0.311) (figure 3*g*; table 1; electronic supplementary material, table S5) in contrast to a strong, albeit not statistically significant, negative effect of variability experienced in the past associated with large sized organisms on SMD (*β*_0_ + *β*_1_ + *β*_6 large_ + *β*_15 large_ = −0.5684), given that the confidence intervals spanned zero (figure 3*h*; table 1; electronic supplementary material, table S5). Similarly, we detected a moderate, yet statistically significant, negative effects of size on SMD for variability experienced in the present (table 1).

In considering additional of features of experimental designs, we discovered weak statistically insignificant positive effects of secondary temperature on SMD, such that with each additional degree of temperature increase, SMD increased by 0.002 (*β*_9_ = 0.0002) (figure 4*e*,*f*; table 1; electronic supplementary material, table S5). Experimental duration was not statistically significant in altering the effect of environmental variation on organisms such that with each additional day experiments were conducted, SMD decreased by 0.047 (*β*_10_ = −0.047) (figure 4*g*,*h*; table 1; electronic supplementary material, table S5), with confidence intervals spanning zero.

## 4. Discussion

Our meta-analysis reveals that thermal variation tends to be detrimental to organismal performance when considered across a broad group of taxa, systems, measured responses and temporal scales. Though a wide range of both positive and negative effect sizes have been reported among the experimental tests of the effects of thermal variation on organismal performance, our analysis suggests that thermal variability tends to precipitate negative effect sizes for acutely exposed organisms and even greater negative effect sizes for organisms previously exposed to variability during an acclimation phase (figure 3*a*,*b*; table 1). Additionally, our results highlight that attributes of the temperature regime, life stage of organisms, and the nature of response metric exacerbate the effects of experienced environmental variation. Thus, both properties of organisms and the structure of thermal variation jointly determine the net effects of thermal variability on biological processes.

Overall, effects of thermal variability on biological responses depend on when thermal variability is experienced (figure 3*a*,*b*; table 1; electronic supplementary material, table S5). Both outcomes (negative effects as a result of variability during acute exposure, and even greater negative effects as a result of variability during acclimation) are not consistent with our first hypothesis that organisms acclimated to thermal conditions will experience benefits relative to non-acclimated organisms. Importantly, due to a paucity of studies that factorially manipulated variation in both experiential phases, there is little experimental evidence to directly evaluate this comparison. These results further highlight the importance of time-dependent responses in the context of environmental variability, for instance critical thermal limits and thermal performance curve shape have been shown to interact with plasticity across a range of time scales [48,49].

Our results additionally suggest the domains over which organisms experience environmental variation can mediate the associated consequences of organisms' performance. We anticipated an observed negative impact of fluctuation range in our second hypothesis, based on the non-linear consequences of disproportionally high environmental temperatures [3–5,11]. However, we observed a positive impact of fluctuation range when organisms experienced variability in the past– with larger amplitude fluctuations producing a significant, albeit subtle, increase in effect size for how variation modifies performance. This outcome could be produced by thermal fluctuations forcing an organism's body temperature closer to the optimal limits of thermal performance than those exposed to single mean temperature [12]. The observed negative impact of mean temperature on the effects of thermal variation indicates that overall, moderate increases in mean temperatures are detrimental to performance, as eventually warmer temperatures precipitate large negative biological effects. Additionally, our finding of simultaneously increasing fluctuation magnitude and mean temperature precipitating significant negative effects further supports previous work that has highlighted that increases in mean temperature are not the sole determinant in negative biological effects [4] and it is likely extreme events that coincide with increased mean temperatures will precipitate negative effects [50]. The context of environmental temperature relative to an organism's physiology is important for understanding how variability may impact performance [51,52]. Given that both mean temperature and variance in mean temperature are anticipated to increase under climate change, organism's ability persist in the face of global change may be challenged depending on the amount of variance, the mean temperature, and when in time variability is experienced.

Our analysis also identifies the importance of traits in mediating the effects of variability, consistent with our third hypothesis. We originally predicted that environmental variation would have a strong negative impact on smaller organisms in an experimental setting, owing to the fact that smaller organisms may be less able to regulate body temperature amidst thermal fluctuations because of their small body mass [24]. Yet our results indicate that organisms of medium size class were associated with strong negative effect sizes for variability regardless of past or present exposure, with small and large sized organisms experiencing weak negative effects of variability in both past and present exposures (figure 3). These findings indicate that regardless of magnitude, organisms experiencing variability in both the past and the present are disadvantaged.

Similarly, our results indicate that organisms across age classes were generally associated with weak negative effect sizes in response to variability when experienced in the present and even weaker negative effect sizes when variability is experienced in the past (figure 3*c–e*; table 1; electronic supplementary material, table S5), which could also be attributed to increases in body mass increasing the ability to buffer variability. This result may be explained by previous work that has shown that in certain cases, thermal tolerance may be modified by life stage, allowing for different mitigation strategies: increased thermal tolerance in older organisms [53], decreased thermal tolerance with age [54], heat shock proteins in buffering effects more significantly in younger organisms [55], and increased plasticity at the cost of a positive effect size, as described by the climate variability hypothesis [56–58].

The data available from experiments testing the effects of thermal variation constitute a highly unbalanced dataset, and our mixed effects modelling approach allowed us to infer effects of variation across this dataset while accounting for fairly extreme differences in the kinds of studies represented (figure 3*a–i*; electronic supplementary material, table S6). The majority of observations in the dataset are from responses measured in acute experimental designs (1227 effect sizes), small sized organisms (1247 effect sizes), juvenile organisms (953 effect sizes), and positive trait directionality (875 effect sizes) out of the total 1712 effect sizes. The mean effect sizes of these groups, when considered alone, are generally positive (figure 3*a-i*). However, our analysis indicates an estimated increase in negative effect sizes in response to variability for these groups (figure 3*a–i*; table 1). The difference between the mean response from the distribution of effect sizes and the modelled estimate reflects the ability of the mixed effects models to account for correlated uncertainties for observations from the same groups (study, phylogenetic group, and response type), and this can counter some of the effects of unbalanced designs [39]. The difference in estimated effect of variation highlights the importance of analytical methods that can account for such uneven representation in the data, and in this case produces a different result (i.e. compare large dots to horizontal lines with 95% confidence intervals in figure 3).

Our analysis reveals that the majority of studies manipulating thermal variation tended to occur over short timescales and that the effects of variability were consistently negative for longer-term experiments (figure 4*g,h*). This was not surprising considering that traits are well established to respond at different rates to thermal conditions [59]. Because the studies included in this analysis report a wide range of biological response types, and certain traits have a faster rate of acclimation than others (e.g. some traits are reversibly plastic within a generation while others fix during development), we anticipated that the experimental duration of both the acclimation phase and acute phase would have influenced the outcome of experienced environmental variation over time. Previous syntheses have emphasized how experimental design in acclimation experiments may be driving different responses across taxa [60], such that species specific acclimation periods precipitate different thermal responses [61] and that beneficial effects of acclimation are only gained when short exposures initially occur [62]. Recent work has also highlighted the importance of traits in predicting when and how organisms may persist in variable environments, via cues or bet-hedging strategies [19]. The preponderance of experiments conducted at relatively short timespans may underestimate the true impacts of variability that occurs in nature over longer timescales such as droughts. Similarly, our findings of weak positive effects of pre-experimental temperature on effect size magnitude further implicates the importance of explicit experimental design choices.

While this meta-analysis provides insight into the nature of the effects of thermal variability, we offer the following caveats regarding the potential limitations of this study. Firstly, because we chose to look at responses across a variety of systems and taxa, we could only draw broad conclusions about the directionality of variability on performance. Our systematic literature search yielded mainly studies on terrestrial ectotherms (*n* = 53, 71% of overall studies), with a reduced amount of aquatic or marine species included (*n* = 22, 29% of overall studies), with marine species being especially underrepresented (*n* = 2, 3% of overall studies). Having reduced studies from marine and aquatic ecosystems may mean a gap in potential strategies organisms use to respond to variability [63]. Future work should also consider the extent to which geographical range modifies the biological response to thermal variation [64]. Our analysis also excluded studies not conducted under controlled laboratory conditions (i.e. *in situ*, observational, reciprocal transplant) in order to control the mean temperature between fluctuating and constant treatments, which may be limiting the variety of responses and subsequent realized effects of natural variability in our analysis. Though studies on thermal variability in terrestrial plants appeared in our search, many were excluded because of their focus on plant responses exclusively to ambient changes rather than controlled fluctuations or interest in thermal extremes but not paired treatments with the same mean temperature. Our analysis included only 25 effect sizes from only 2 studies involving terrestrial plants as a result of this. We can therefore only speculate about the extent to which observations from the controlled laboratory experiments analysed here represent responses to thermal variability in more complex, natural environments.

Additionally, all studies included in this analysis featured diurnal fluctuations in temperature due to limited studies manipulating aspects of thermal variation besides total variance (e.g. temporal autocorrelation [65]), which are known to exert additional biological effects such as more positively autocorrelated (reddened) temperature time series that are conducive to heightening inflationary effects in environments with immigration [2,7]. As additional empirical studies on such effects become more numerous, future meta-analyses could target ecologically complex and realistic patterns of variability, as conclusions from this work will best inform an understanding of the effects of sinusoidal variability patterns. Finally, because few studies in our analysis quantified population or community-level responses to variability across experimental designs (figure 2), opportunities exist for future research to uncover how higher levels of biological organization respond to thermal fluctuations and previous exposure to thermal fluctuations.

Thermal variability has become an increasingly important focus of global change biology as the recent regional patterns of variability shift [7] and impact biological processes [4,66]. Alterations to Earth's historical patterns of variability can disrupt biological processes and the results of this meta-analysis demonstrate that biological responses to variation are highly context-dependent, including both positive and negative consequences. Our results indicate that important components of life history, traits, and environment can exacerbate the effects of thermal variability and that ultimately, variability is not universally beneficial or detrimental. Continuing to investigate the mechanisms and drivers of responses to thermal variability across levels, organisms, and timespans remains an important goal for global change ecology research.

## Data Availability

Data are archived on Dryad. All code and data for analyses are available at http://dx.doi.org/10.5281/zenodo.7545496 [67]. The data are provided in electronic supplementary material [68].
